# Genomic Analysis of the Recent Viral Isolate vB_BthP-Goe4 Reveals Increased Diversity of φ29-Like Phages

**DOI:** 10.3390/v10110624

**Published:** 2018-11-13

**Authors:** Tobias Schilling, Michael Hoppert, Robert Hertel

**Affiliations:** 1 Department of Genomic and Applied Microbiology & Göttingen Genomics Laboratory, Institute of Microbiology and Genetics, Georg-August-University Göttingen, 37077 Göttingen, Germany; tschill2@gwdg.de; 2 Department of General Microbiology, Institute of Microbiology and Genetics, Georg-August-University Göttingen, 37077 Göttingen, Germany; mhopper@gwdg.de

**Keywords:** *Bacillus*, thuringiensis, vB_BthP-Goe4, Goe4, Picovirinae, Phi29virus, *Salasvirus*, Luci, bacteriophage, phage, pRNA

## Abstract

We present the recently isolated virus vB_BthP-Goe4 infecting *Bacillus thuringiensis* HD1. Morphological investigation via transmission electron microscopy revealed key characteristics of the genus *Phi29virus*, but with an elongated head resulting in larger virion particles of approximately 50 nm width and 120 nm height. Genome sequencing and analysis resulted in a linear phage chromosome of approximately 26 kb, harbouring 40 protein-encoding genes and a packaging RNA. Sequence comparison confirmed the relation to the *Phi29virus* genus and genomes of other related strains. A global average nucleotide identity analysis of all identified φ29-like viruses revealed the formation of several new groups previously not observed. The largest group includes Goe4 and may significantly expand the genus *Phi29virus* (*Salasvirus*) or the *Picovirinae* subfamily.

## 1. Introduction

Bacteriophages or phages are viruses of bacteria and probably the most common biological entities on earth. Phage species outnumber their hosts by 10 times [1] and thus, represent the largest unexplored genetic reservoir. Bacteriophages play an essential role in the control of prokaryotic populations, their biomass turnover and their diversification. In addition to the ecological effects, phages also have economic relevance. Unintentional contamination of industrial fermenters can destroy entire productions. Molecular biology would not be the same without phages or selected phage proteins. Even the current genomic revolution, driven by the prospects of CRISPR-Cas, is related to phages, as CRISPR-Cas is a bacterial phage defence system [2]. Currently, phages are in the focus of medicine. For example, the first centre in USA for phage-based treatment methods for antibiotic-resistant bacteria was recently opened [3]. Therefore, it is important to further explore phage diversity in order to take advantage of all options.

In the current study, we used a *Bacillus* strain as the host for recovering novel phages from the environment. The genus *Bacillus* represents Gram-positive, aerobic, endospore-forming bacteria and incorporates many species that inhabit soil [4]. It includes species whose activity for humans can be either negative or positive. For instance, *B. anthracis* causes anthrax and is therefore a serious pathogen. Its closely related species *B. thuringiensis* is harmless to humans and is frequently used as a biological control agent in agriculture. *B. subtilis* is a well-established model organism of molecular biology [5] and led as host for phage isolation to the discovery of many important viral strains e.g., φ29 [6].

Phages of the φ29-like group are the smallest *Bacillus* infecting phages (for review see [7]) and members of the *Podoviridae* family. The sequenced representatives harbour a small linear genome (18.4–21.1 kbp [8]) covalently linked to a terminal protein (TP) at the 5′ genome end. A DNA replication mechanism first investigated in this phage group has attracted particular attention: Protein-primed genome replication, is initiated at the origin at the 3′ end of the genome together with a TP protein by forming a TP-dAMP complex, using the second base of the template. Afterwards a slide back mechanism to the first base of the template takes place and the second base serves again as template. The genome termini of φ29-like phages show inverted terminal repeats (ITRs), which are essential for the initiation of genome replication and until now always revealed three identical nucleotides in the last three positions, making a slide back possible without loss of information. After binding of the first two bases, a short 10 bp fragment is synthesized and the DNA polymerase is released. This TP-10 bp primer allows the DNA polymerase to reattach to complete genome synthesis. The monomeric B-type DNA polymerase catalysis both the initialization and elongation of DNA synthesis. The DNA polymerase shows 3′-5′ exo-nucleolysis activity, which enables proofreading and strand displacement, making accessory proteins like DNA helicases unnecessary. These features make the φ29 DNA polymerase an important tool for multiple displacement DNA amplification, in particular for long read lengths in genome amplification [9]. Another feature appears to be unique to the φ29 phage: A translocation machinery consisting of the packaging RNA (pRNA), the upper collar protein (gp10) and the DNA encapsidation protein (gp16) form a unique motor that, driven by ATP hydrolysis, efficiently translocate the viral genome into the head of the phage [10]. In view of phage-host interactions, several key events in φ29 phage development have been elucidated in detail: The defence mechanism of the phage against the host’s uracil excision activity [11], and the involvement of the *Bacillus* MreB protein in viral development [12]. Consequently φ29-like viruses are resources for novel enzymes, useful for applications in molecular biology (namely, amplification of DNA) and provide important insights into evolution of phage assembly and host interaction mechanisms. Currently the genus *Phi29virus*, harbouring the type strain φ29, is in the transition to be renamed into *Salasvirus* [13] in honour of Margarita Salas who has worked on φ29 for over 50 years.

In this study, we present the bacteriophage vB_BthP-Goe4 (Goe4) isolated with *Bacillus thuringiens* HD-1 [14] as host. Its morphology and genome sequence were characterized, and comparative analysis of related strains revealed new groups of φ29-like phages.

## 2. Materials and Methods

### 2.1. Phage Isolation and Genome Sequencing

The virus vB_BthP-Goe4 was isolated from the Göttingen municipal sewage plant (Göttingen, Germany, 51°33′15.4″ N 9°55′06.4″ E) via an overlay plaque assay using *Bacillus thuringiens* HD-1 [14] as the host. Culture conditions and overlay techniques were applied as described previously [15].

Phage genomic DNA was prepared with the MasterPure complete DNA and RNA purification kit (Epicentre, Madison, WI, USA). Paired-end Illumina sequencing libraries were generated with the Nextera XT DNA sample preparation kit and were sequenced with a MiSeq instrument and MiSeq reagent kit v3 as recommended by the manufacturer (Illumina, San Diego, CA, USA). Trimming and quality-filtering of the recovered reads was performed with Trimmomatic version 0.36 [16] and analysed with FastQC version 0.11.7 [17]. Initial assembly was performed with SPAdes version 3.12.0 [18] using 394,938 high-quality paired reads as input and resulted in a single contig with a coverage of 2,900,000. Genome ends were verified via Sanger sequencing as described previously [15] with the primers PP005 (5′ GTTTGTTACTGTTCTGCGTTTAGC) and PP006 (5′ CCGACAAATGGTGGGTACTG). Initial ORF (open reading frame) calling and automatic annotation was done with the Prokka pipeline [19] with implemented pVOG database [20]. Identified protein-encoding genes were compared using the web based NCBI blastp and a global alignment with the φ29protein (see below). All hypothetical proteins were additionally verified for the presence of known domain structures by employing InterProScan [21]. The final annotated genome sequence of vB_BthP-Goe4 was submitted to GenBank and is publicly available under the accession number MH817022. Biological samples of vB_BthP-Goe4 are available from the German Collection of Microorganisms and Cell Cultures (DSMZ) under the sample number DSM105107.

### 2.2. Transmission Electron Microscopy

Transmission electron microscopy (TEM) analysis were performed as described previously [15,22]. Phosphotungstic acid dissolved in pure water (3% *w*/*v*) and adjusted to pH 7.0 served as staining solution. Electron microscopy was carried out on a Jeol 1011 electron microscope (Eching, Munich, Germany) at calibrated magnifications. Calibrations were performed with a cross grating replica, with rulings of 2160 lines/mm according to manufacturer’s instructions (Plano, Wetzlar, Germany). Images were captured using a Gatan Orius 4 K camera and processed with the Gatan 314 Digital Micrograph software package (Gatan, GmbH, Munich, Germany) and Adobe Photoshop CS2 (Adobe Systems Inc., Jan José, CA, USA). The average sizes of each isolate based on results obtained from of at least six individual virions.

### 2.3. Genome Analysis and Comparison

Global genome alignment of Goe4 and φ29 on nucleotide level was done with EMBOSS stretcher service of the EBI website [23]. Deduced protein sequences of phage φ29 and Goe4 were compared using blastp [24]. The resulting protein pairs were globally aligned with Needleman-Wunsch-algorithm using the needle program of EMBOSS suite [25] with the default parameters. Promoter prediction was performed with PePPER (http://genome2d.molgenrug.nl/g2d_pepper_promoters.php) [26]. The prediction of rho independent transcription terminators was made with TransTermHP 2.08 [27] and ARNold (http://rna.igmors.u-psud.fr/toolbox/arnold/index.php) [28,29] on an un-annotated Goe4 genome sequence. Pairwise phage genome comparison was done with tblastx [24], visualized with Easyfig 2.2.2. [30]. Data output of both comparisons were combined and further processed with Adobe Illustrator CS2 (Adobe Systems Inc., San José, CA, USA).

Average nucleotide identity (ANI) [31] was calculated using the average_nucleotide_identity.py (https://github.com/widdowquinn/pyani) script with the ANIm option employing MUMer3 [32] and the ANIb option employing blastn [24] for global genome alignments. The pRNA in the genome of Goe4 and its close relatives were indentified by using the Infernal 1.1.2 software package [33]. The required co-variance model was calculated using the pRNA gene sequences from virus Nf (EU622808.1) and GA-1 (NC_002649.1) as input and is included in the supplementary (pRNA-Nf-GA1.cm). Both pRNA sequences were previously aligned with the LocARNA web tool [34] with default parameters and saved in Stockholm format.

GC-contents of all phage genomes were calculated using the script get_gc_contetn.pl by Jennifer Meneghin [35].

To identify the potential host for phage Luci, a blastn search with the *Lucilia cuprina* draft genome (accession number JRES00000000) against the non-redundant NCBI database was performed, and second best hits were evaluated to avoid a self-hit (Table S1).

### 2.4. Orthology and Evolutionary Analyses

Protein sequences were extracted from GenBank files using the cds_extractor.pl script (http://dx.doi.org/10.5281/zenodo.215824). All protein sequences were used for orthology analysis employing Proteinortho V5.16b [36]. Evolutionary analyses were conducted in MEGA7 [37]. Initially 23 DNA-polymerase amino acid sequences and 22 pre-neck-appendage protein amino acid sequences were aligned with the MUSCLE [38] using default parameters suggested by MEGA7. From this alignment phylogeny was reconstructed using the maximum likelihood method based on the JTT matrix-based model [39] and tested with the bootstrap method with 500 replications. All sites from the alignment were used for calculation. Search for an optimal tree structure was done with the nearest-neighbour interchange method.

## 3. Results

### 3.1. Isolation and Morphological Characterisation of vB_BthP-Goe4

**Figure 1:**
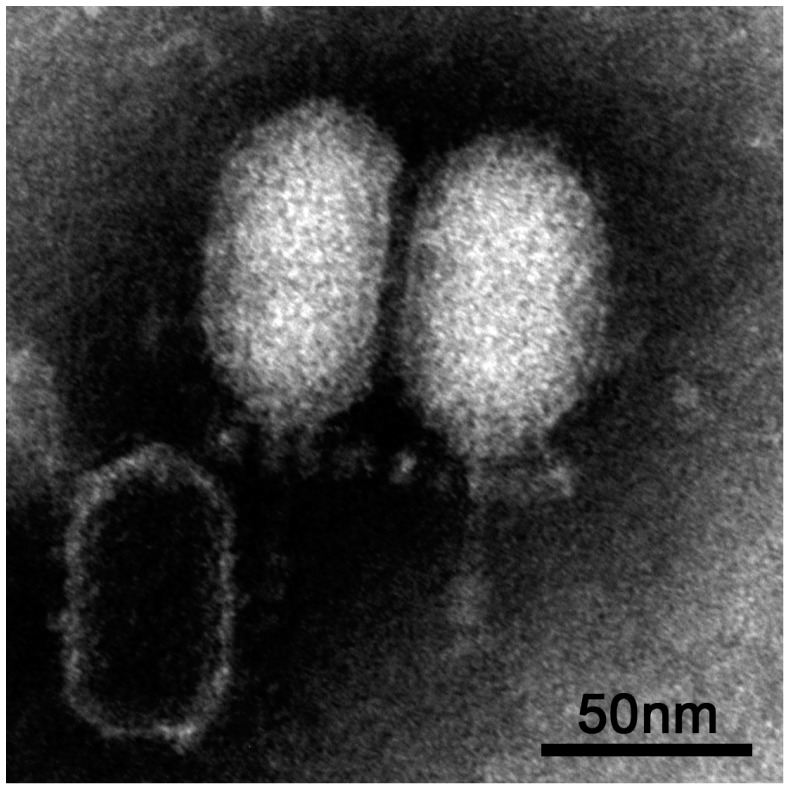
vB_BthP-Goe4 Micrograph.

The virus vB_BthP-Goe4 (Goe4) was isolated from raw sewage using *Bacillus thuringiens* HD-1 [14] as host bacterium. TEM analysis revealed a head-tail structure, which is typical for members of the order *Caudovirales* (see Figure 1). An elongated head (height 70.7 nm ± 1.9 nm and width 50.4 nm ± 1.5 nm) and a short non-contractile tail (length 45.4 nm ± 2.8 nm and width 6.6 nm ± 0.4 nm) allowed a classification into the *Podoviridae* virus family, whereas its dimensions and tail structure (Figure 1) indicated an association with the *Picovirinae* subfamily [40].

### 3.2. Genomic Characterization of vB_BthP-Goe4

**Figure 2:**
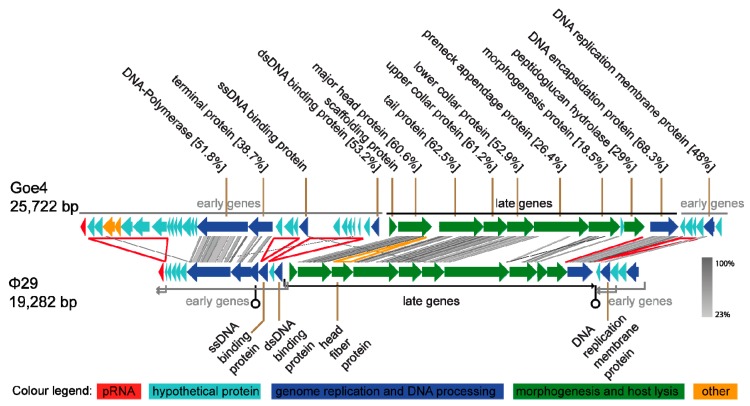
Overview of the Goe4 genome in Relation to φ29. Similarity indicated by grey bars was calculated via tblastx. Protein coding open reading frames (ORFs) were further compared to the genus type strain φ29 via global protein alignment employing the Needleman–Wunsch algorithm. The yellow arrowhead indicates a region of φ29 absent in Goe4 and red arrowheads and line regions of Goe4 absent in φ29.

Genome sequencing and annotation revealed a linear 25,722 bp viral chromosome with 30.43% GC content. The genome encoded one ncRNA packaging RNA (pRNA) and 43 putative proteins of which 16 could be assigned with a potential function. Annotated genes showed frequent similarity to genes of *B. subtilis*-infecting virus φ29 (NC_011048). Direct comparison of Goe4 and φ29 genomes revealed a high degree of similarity with respect to genome organisation and gene content (Figure 2). Approximately 80% of the φ29 genome components showed similarity to the corresponding ones in the genome of Goe4. Highest identities were recorded for the genes involved in morphogenesis and genes encoding the DNA-polymerase and the terminal protein located in the early region on the left genome site. These homologies allowed classification of Goe4 into the φ29-like virus group. Due to the genome composition of Goe4 compared with φ29 we postulate two early regions, at the left and right genome ends of Goe4, and a late region in the genome center (Figure 2). The unique genes of Goe4 are encoded by the early regions, which are variable among φ29 and its closer relatives [8]. Global genome alignment of Goe4 with φ29 revealed 43.4% nucleotide sequence similarity, thereby indicating that Goe4 is not a species within the *Phi29virus* genus and may open up a new genus within the *Picovirinae* subfamily.

In addition to the variations in the early regions, differences could be observed in the conserved late region. The first is located between the major head protein gene and the tail protein gene (Figure 2, yellow arrowhead). In φ29, the head fibre gene is located at this position. The second region is located between the peptidoglucan hydrolase gene and the gene of the DNA encapsidation protein (Figure 2, red line). Both regions might be involved in regulatory processes. At the first region, rho-independent transcription terminator was predicted without a promoter region for the transcription initiation of the downstream genes. At the second region, which showed a strong drop in GC-content upstream of the coding region of the DNA encapsidation protein, a promoter was predicted (Supplemental Table S2).

The organization of the early genomic regions of Goe4 is different from its φ29 counterparts. Two genes are missing in the right early region, including early protein 17, which is involved in replication, but is non-essential [41]. The size of the right early region is similar between the two phages, due to the presence of new Goe4 specific genes (see Figure 2).

The left end early region reveals more variations and compared with phage φ29 harbours 16 additional protein-coding genes, arranged in three sets (see Figure 2, red arrowheads). The first is located between the pRNA and a series of short hypothetical proteins genes, the second between the terminal protein and the ssDNA binding protein gene and the third between the ssDNA binding protein and a hypothetical protein gene in front of the dsDNA binding protein gene. Almost all Goe4-specific genomic elements contain hypothetical proteins. The genomic element near the left genome border harbours seven large protein-encoding genes of which five are hypothetical and two were assigned as potential dUTPase/dCTP pyrophosphatase and glutaredoxin.

### 3.3. Goe4 and Its Closest Relatives

A global blastn analysis using the Goe4 genome as query against the non-redundant nucleotide data base of NCBI resulted in the identification of nine closely related virus strains (Juan (MF156577), RadRaab (MF156580.1), Stich (KX349901.1), KonjoTrouble (MF156578.1), Aurora (KX349899.1), QCM11 (KX961631.1) [42], SerPounce (KY947509.1) [43], Claudi (KX349900.1) and MG-B1 (KC685370.1) [44]). Many of those employ *B. thuringiesnis* as host, but also other members of the *B. cereus* clade [45] like *B. weihenstephanensis* [44], phage QCM11 in *B. anthracis* [42]. Members of this bacterial clade are genomically conserved and mainly differ in number and content of plasmids, which define their lifestyle [45]. To identify also distinct relationships, the nucleotide sequences of the Goe4 DNA polymerase and the terminal protein were used as query for a blastx search against the NCBI nr database. In this way, two more *B. thuringiensis*-infecting phages BeachBum (KY921761.1) and Harambe (KY921761.1) [43], the known *Phi29virus* genus members [8], a φ29-like virus VMY22 (KT780304.1) [46] and a contig Scaffold4203 (JRES01001113.1) derived from the draft genome sequence of *Lucilia cuprina* [47] were identified. Automatic re-annotation of contig Scaffold4203 from Australian sheep blowfly *L. cuprina* (16,580 bp) resulted in a virus genome similar to φ29, but lacking the early genes at the right end of the genome. Due to its origin, this virus was named Luci (see supplemental material for the artificial GenBank file Luci_art.gbk) and included in the analysis. It is known that the genus *Lucilia* is associated with many *Firmicutes* [48] so it can be expected that Luci also replicates on a *Bacillus* host. Blastn analysis of the *Lucilia cuprina* draft genome [47] against the non-redundant database of NCBI revealed a scaffold contig JRES01000365.1 being similar to *Paenibacillus polymyxa*. The resemblance was only very slight, but supports the previous assumption (Table S1).

**Figure 3:**
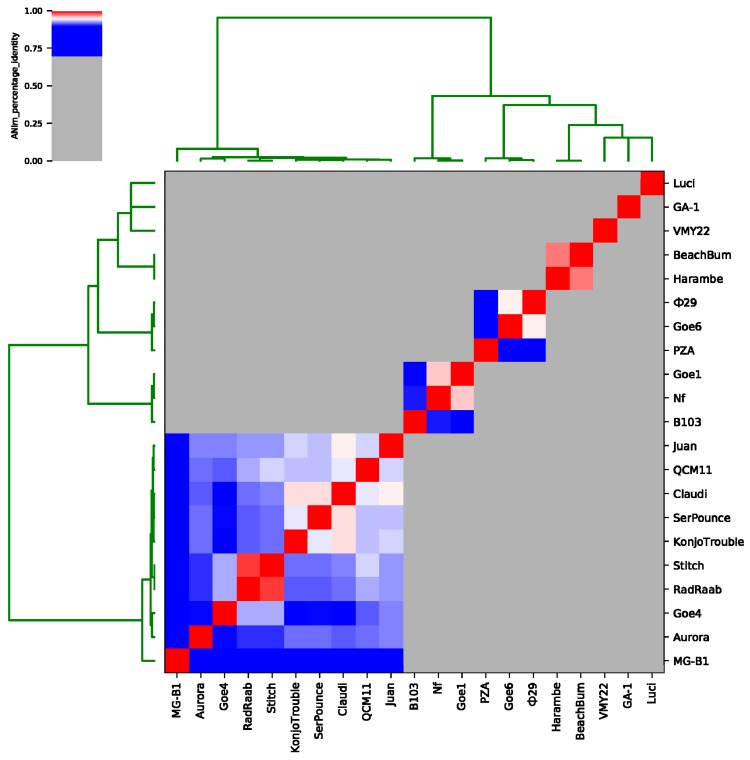
Average nucleotide identity analysis of Goe4 with twenty related strains. The presented average nucleotide identity values were calculated with the ANIm option. Reddish colouring indicates average nucleotide identity (ANI) values >95% and belonging to the same species. A white coloration indicates ANI values of ~95% and the species boundary. A bluish coloration indicates ANI values <95% to 70% and a high degree of relationship.

To expose relationships of Goe4 to its above-identified closest relatives, average nucleotide identity (ANI) values were calculated (see Figure 3 and Table S5). A separation in seven sequence clusters was recorded. Goe4 groups together with MG-B1, Aurora, RadRaab, Stich, KonjoTrouble, SerPounce, Claudi, QCM11 and Juan, thereby revealing highest similarity to phages Stich and RadRaab with a ANI value of approximately 93%. RadRaab and Stitch share an ANI value of 98%, which is indicative for members of the same species [49]. Furthermore, phage Claudi showed an ANI value of ≥95% in relation to SerPounce and KonjoTrouble. Unfortunately, the assignment of the three phages to the same species is not possible, since analysis of SerPounce and KonjoTrouble did not show an ANI value of ≥95%. The most distant member of this cluster is MG-B1, which matches best with KonjoTrouble (ANI value approximately 85%). The remaining phages split in six more clusters. The first one comprised phage Nf (EU622808.1), B103 (X99260.1) and Goe1 (KU831549.1) [8], the second one φ29 (EU771092.1), PZA (M11813.1) and Goe6 (MF407276.1) [50], third one Harambe, BeachBum and three more with only one representative, like VMY22, GA-1 (X96987.2) [51] and Luci. Goe1 and Nf seem to be of the same species (ANI value 96%), as well as φ29 and Goe6 (ANI value 95%), and BeachBum and Harambe (ANI value 97%).

**Figure 4:**
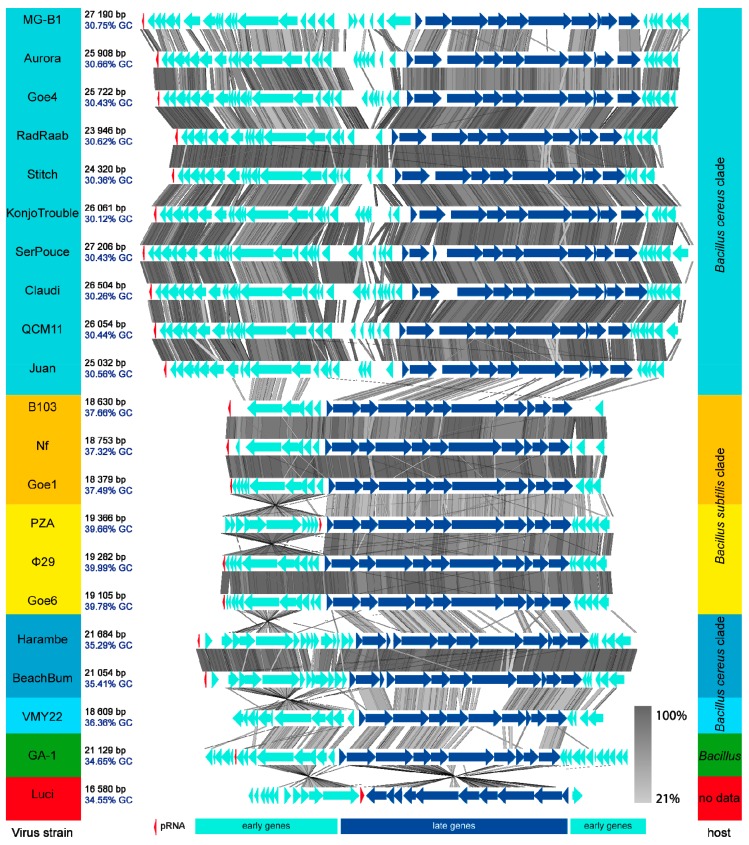
Blast Comparison of all φ29-like Phages. Genomes are ordered like presented in the ANI analysis in Figure 3. Phages are compared via tblastx using the Easyfig tool [30]. Name, genome size and GC-content of each phage are listed on the left side and highlighted in colour. Members of the same cluster are equally highlighted. The respective hosts are listed on the right side. Bluish highlighted strains infect hosts of the *B. cereus* clade, yellowish highlighted strains infect hosts of the *B. subtilis* clade. pRNA genes are shown as red arrows and were identified via a covariance model created during this investigation (supplemental pRNA-Nf-GA1.cm).

The results of global tblastx of each phage with its respective closest relatives, reveal an overall consistent genome organisation of all viruses (Figure 4). The GC-content correlates with the cluster formation observed during the previous ANI analysis (see Figure 3). A non-coding genomic region of Goe4 between the genes encoding the major head protein and the tail protein (see Figure 1) is frequently observed among most members, but with variable size. Phage MG-B1 is special as it lacks this intergenic region. Early gene sets are generally of higher diversity with strain-specific areas at the right end of the left early regions. Phages related to the clusters harboring phage B103 and φ29 are very similar with conserved genome synteny. Phage B103-associated genomes are shorter and of lower GC-content compared to those associated with φ29. Phage Harambe and BeachBum both revealed an opposite orientation of their left early region with respect to the majority of other phages and may thereby represent a specific property of this phage group. VMY22, GA-1 and Luci remain single. The most characteristic features of these three unique phage genomes are size, genomic organisation and position of the pRNA (see Figure 4). In frame of this investigation, the pRNA was bioinformaticaly predicted in all phage genomes, beside phage VMY22 (Table S3). In almost all strains it is found at the end of the left early gene region. GA-1 is the only genome revealing an additional operon on the left border downstream the pRNA gene. Due to the draft status of the genome Luci the predicted position of the pRNA was not considered for this analysis.

To further examine the relationship among the present φ29-like goupe members, we focused on the inverted terminal repeats (ITRs), which are essential for protein primed initiation of genome replication [7]. All in all, 13 of 21 phage genomes clearly exhibited their ITRs at their genome ends (Table S4). The ITRs were of five different types and correlated with the observed clustering of the phages. All members of the Goe4 cluster harbor a conserved 7 bp core ITR (5′ AAATGTA), with the only exception of phage MG-B1, which revealed a specific 7 bp ITR (5′ AAATATA). Members of the clusters with phages Nf, Goe1, B103, Goe6 and φ29, showed a 6 bp long conserved core ITR (5′ AAAGTA). Phage Harambe and BeachBum revealed the longest ITR with approximately 16 bp (5′ AAGATAGCCCCCCACC) and the first with only two identical nucleotides at its 5′ end. Phage GA-1 revealed the last type of ITR. It is 7 bp in size and its sequence (5′ AAATAGA) differs from the MG-B1 ITR only in one base. No ITRs could be identified for phage VMY22 what indicates its draft status. Of the phages that infect *B. subtilis*, only PZA showed no ITRs at either genome end. However, the right end of the genome showed the respective AAAGTA sequence, which corresponds to the phages related to B103 and φ29. The absence of the ITR at the left end of the genome seems to be the result of an incorrect assembly, which is indicated by the twisted left genome region (see Figure 4). In such a case, the ITR would orientate itself towards the genome centre. In fact, such an ITR hexamer can be found between the pRNA gene of PZA and its scaffold protein gene (positions 5195–5200). The distance of 141 bp between pRNA gene and ITR is the same as in φ29 and Goe6, which supports the assumption of an assembly artefact.

### 3.4. Orthology and Evolutionary Analyses

To find out about common proteins among all investigated and obviously related phages and to describe group specific proteins we performed an orthology analysis. Beside all φ29 related phages, the *Streptococcus* phage Cp-1 [23], a non-*Bacillus* infecting *Picovirinae* and the membrane-containing virus PRD1 infecting *Gammaproteobacteria* [52] were introduced to this analysis. The last mentioned replicates its genome similar to φ29 [52]. The results revealed the DNA-polymerase as the only orthologue protein among all investigated viruses. Cp1 shared three more orthologue proteins with the other *Picovirinae*, like the DNA encapsidation protein, upper collar protein and the major head protein. The phage group associated with Goe4 revealed common orthologues like the dUTPase/dCTP pyrophosphatase, Glutaredoxin, ssDNA binding protein, dsDNA binding protein and four hypothetical genes from the left early gene region, the first hypothetical gene from the late gene region and the last hypothetical gene from the right early gene region. Joint orthologues with phage BeachtBum and Haramba could also be observed. These two phages in turn also shared specific orthologues with the remaining strains outside the Goe4 group. For more details, consult the primary results present in the supplemental Table S6.

**Figure 5:**
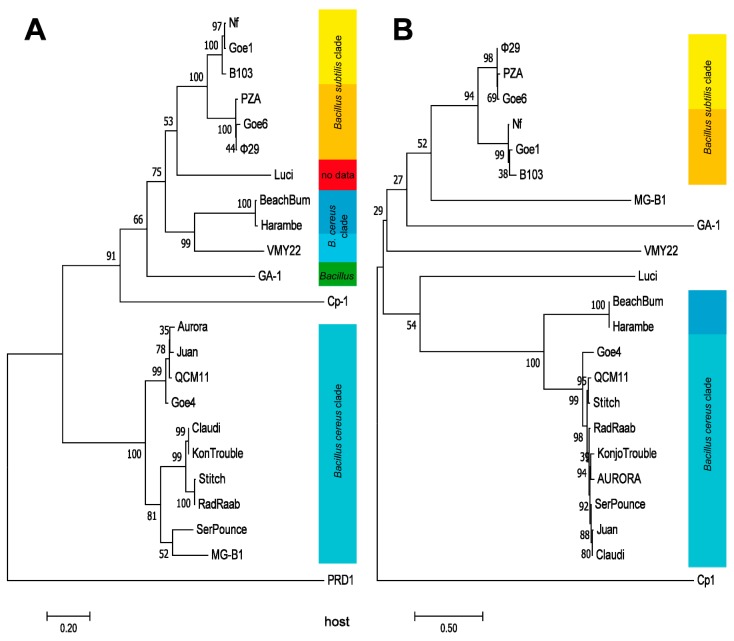
Molecular Phylogenetic analysis, using the Maximum Likelihood method. The evolutionary history was inferred by using the Maximum Likelihood method based on the JTT matrix-based model [39]. Trees with the highest log likelihood (−13,127.87 for (**A**) and −17,734.54 for (**B**)) are shown. The percentage of trees in which the associated taxa clustered together are shown next to the branches. Initial tree(s) for the heuristic search were obtained automatically by applying Neighbor-Join and BioNJ algorithms to a matrix of pairwise distances estimated using a JTT model, and then selecting the topology with superior log likelihood value. The trees are drawn to scale, with branch lengths measured in the number of substitutions per site. DNA-polymerase served as marker protein for tree (**A**) and the pre-neck-appendage protein for (**B**). The analysis involved 23 amino acid sequences with a total of 735 positions in the final dataset for (**A**) and 22 amino acid sequences with a total of 1015 positions for (**B**). The respective hosts are listed on the right side of each dendrogram and colour coded like in Figure 4.

A marker protein based phylogeny was calculated in order to further clarify the evolutionary relationships of the individual viruses. The DNA polymerase was predestined for this calculation, due to its presence as conserved proteins in all examined *Picovirinae* and in the outlier PRD1. The resulting dendrogram, shown in Figure 5A, revealed a very similar grouping like seen on the average nucleotide identity analysis (see Figure 3). Phages associated with Goe4 organized again in a similar manner and evolutionarily split off even before the phage Cp1. Phages BeachBum, Haramba and VMY22 are closer associated with φ29 related phages than with those associate with Goe4, even they also infect bacteria of the *B. cereus* clade. Thus, all three are new representatives of the genus *Phi29virus*. A further dendrogram, was calculated using the pre-neck-appendage protein (Figure 5B). With respect to phages BeachBum and Haramba the observed results were contradictive to the previous ones (Figure 3 and Figure 5A) and showed a host-related grouping with the Goe4 clade. This means that proteins involved in host interaction are not suitable for phylogenetic analysis.

## 4. Discussion

The comparison of Goe4 with closely related strains showed a conserved genetic structure among all phages. The late genome region, which mainly contains the structural genes, is particularly conserved (Figure 4). The scaffolding protein, located in this region, is distinct from the corresponding one of φ29 (Figure 2). This protein is essential for the maturation of the prohead [53]. The head is the main morphological difference between Goe4 and φ29 (elongated and without head fibres). This is supported by the orthology analysis, revealing two distinct conserved proteins (Table S6). The pre-neck appendage protein and morphogenesis protein needed for tail assembly [54] differed strongly from their counterparts in φ29 with protein similarities below 30% (Figure 2). Pre-neck appendage proteins of both phages contain a predicted pectin lyase fold with a beta-helix repeat. Investigation on a very similar protein Dpo7 from phage vB_SepiS-phiIPLA7 infecting *Staphylococcus epidermidis* showed depolarization activity and was successfully used for biofilm degradation [55]. Furthermore, the pre-neck appendage protein of φ29 participates in host cell recognition and entry whereas the morphogenesis protein of φ29 is involved in cleavage of both the polysaccharide backbone and peptide cross-links of the cell wall during infection [56]. Thus, the potentially different exopolysaccharide composition of the *B. thuringiensis* and *B. subtilis* host cells, explains the differences at gene level. This hypothesis is supported by the fact that BeachBum and Harambe, which are closely related to φ29 according to the presented analyses (Figure 3 and Figure 5A), harbour pre-neck appendage proteins with high similarity to those of the Goe4 group (Table S6). In line are also the different pre-neck appendage proteins of phages VMY22, GA-1 and Luci (Figure 5B and Table S6), as each replicates on a distinct bacterial species or genus (potentially cold active *B. cereus* for VMY22 [46], *B. pumilus* for GA-1 [57], *Paenibacillus* for Luci).

The left early region of Goe4 is much larger, with respect to φ29, and shows an increased dissimilarity. The question arises which purpose the many additional hypothetical genes might serve. From phages φ29 it is known that genes of this region are involved in host interaction, as for example gp56 is involved in inhibiting uracil DNA glycosylase and thus preventing host factors from interfering with phage replication [58]. Now, the genomes of *B. thuringiensis*, and of other representatives of *B. cereus* clade, are about 1 Mbp larger (~+25%) than of *B. subtilis*, resulting in a more diverse enzymatic equipment, which the phage must handle to ensure its replication. It might be conceivable that the additional viral genes of the left early region are present due to this fact. Supportive indications for this assumption came from the smallest *Phi29virus* vB_BsuP-Goe1 (Goe1) which has been isolated recently [8]. The fact that this phage was isolated just now and not decades earlier is probably related to its host, which is a genome-reduced mutant (~−8%) of the model organism *B. subtilis* 168 [59]. Phage Goe1 can replicate only very poorly on *B. subtilis* 168 [8]. Probably, it lacks the equipment to tame the extra genes of this strain in relation to its isolation host. The deletion of further genes from the left early region of Goe1 without obvious reproduction limitation on its genome-reduced host further support this assumption [60].

According to the Bacterial and Archaeal Viruses Subcommittee (BAVS), members of the same genus shall share >50% nucleotide sequence similarity [49]. With that, we can state that all strains associated within the Goe4 group are members of the same genus. Now the question arises whether this group should be assigned to the existing *Phi29virus* genus or whether they should open up a new. Arguments for the implementation into *Phi29virus* could be the common genomic organization of both groups. Thus, many genome components of φ29 (~80%) can be found in Goe4. In addition, the pRNA of all Goe4 associated strains could be identified with a covariance model based on pRNA sequences of current *Phi29virus* members. This indicates that the genome translocation system of Goe4 is very closely related to the one of φ29. The same covariance model could not reliably identify the pRNA of Cp1 [61], another *Picovirinae*, but not a *Phi29virus*. Arguments for the establishment of an own genus would be the low nucleotide based similarities of Goe4 and φ29 and the ~35% larger genome of Goe4, which consequently brings with it a complex and diverse constituents, so far not observed within the genus *Phi29virus*. In addition, phylogenetic analysis (Figure 5A) showed that Goe4 associated viruses all group together and that this cluster evolutionary branches off even before the phage Cp1, a representative of a separate genus *Cp1virus*. Therefore, the question whether Goe4 forms a new genus or whether to integrate it into the existing *Phi29virus* is not closed and needs further discussion.

## Supplementary Materials

Supplementary materials can be found at http://www.mdpi.com/1999-4915/10/11/624/s1, Luci_art.gbk; pRNA-Nf-GA1.cm; Table S1: Luci host search; Table S2: Promoter terminator prediction; Table S3: pRNA prediction; Table S4: ITRs of φ29-like-phages; Table S5: ANI percentage identity; Table S6: Orthology analysis.
